# Data in support of peptidomic analysis of spermatozoa during epididymal maturation

**DOI:** 10.1016/j.dib.2014.10.003

**Published:** 2014-11-06

**Authors:** Valérie Labas, Lucie Spina, Clémence Belleannee, Ana-Paula Teixeira-Gomes, Audrey Gargaros, Françoise Dacheux, Jean-Louis Dacheux

**Affiliations:** a INRA, Plateforme d’Analyse Intégrative des Biomolécules, Laboratoire de Spectrométrie de Masse, F-37380 Nouzilly, France; b INRA, UMR85 Physiologie de la Reproduction et des Comportements, F-37380 Nouzilly, France; c CNRS, UMR7247, F-37380 Nouzilly, France; d Université François Rabelais de Tours, F-37000 Tours, France; e IFCE, F-37380 Nouzilly, France; f INRA, UMR1282 Infectiologie et Santé Publique, F-37380 Nouzilly, France; g Université François Rabelais de Tours, UMR1282 Infectiologie et Santé Publique, F-37000 Tours, France

**Keywords:** Peptidome, Spermatozoa, Maturation, Epididymis

## Abstract

The final differentiation of the male germ cell occurs in the epididymal duct where the spermatozoa develop the ability to be motile and fertilize an ovum. Understanding of these biological processes is the key to understanding and controlling male fertility. Comparative studies between several epididymal maturation states could be an informative approach to finding sperm modifications related to maturation and fertility. Here we show the data from differential peptidomic/proteomic analyses on spermatozoa isolated from 4 epididymal regions (immature to mature stage) using a profiling approach based on MALDI-TOF mass spectrometry and, combined to top-down MS in order to identify peptidoforms and proteoforms. By this way, 172*m*/*z* peaks ranging between 2 and 20 kDa were found to be modified during maturation of sperm. A total of 62*m*/*z* were identified corresponding to 32 different molecular species. The interpretation of these data can be found in the research article published by Labas and colleagues in the Journal of Proteomics in 2014 [1].

## Specifications table

**Table:** 

Subject area	Reproductive biology

More specific subject area	Peptidomic analysis of boar epididymal spermatozoa and molecular phenotypes at different maturation stages
Type of data	*Top down raw data and.csv tables with identified proteins*
How data was acquired	*Experiments performed on a LTQ Orbitrap Velos Mass Spectrometer (Thermo Fisher Scientific, Bremen, Germany)*
Data format	*Raw data*
	*.csv results tables of top-down identification*
Experimental factors	*Boar spermatozoa from 4 different regions of the epididymis*
Experimental features	*Epididymal spermatozoa peptidome* (*Sus scrofa*) *and investigation of the boar sperm maturation process*
Data source location	*N/A*
Data accessibility	*Data are available here and via the PRIDE partner repository with the dataset identifier PXD001303.*

## Value of the data

• First description of peptidome/degradome of epididymal spermatozoa from boar (*Sus scrofa*).
• Molecular phenotypes distinguishing degrees of maturation of spermatozoa during the epididymal transit.
• First description of protease activities involved in maturation of epididymal spermatozoa.

## 1 Data

Mean peak values obtained from intact cells (IC), detergent-soluble extracts (SD) and detergent-insoluble extracts (ID) associated with immature (IC2, SD2, ID2) and mature epididymal spermatozoa (IC9, SD9, ID9) are shown in Supplementary Table 1. Only 172*m*/*z* peak values for which the fold-changes were >2 and which presented at least one significant difference between epididymal samples (*p*<0.05) are reported in this table. Some of them are identified. The variation index was calculated as the product of the absolute difference between the maximum (max) and minimum (min) peak intensity values multiplied by the fold-change (fold) between the two extreme values. The variations are expressed as linear decrease (LD) or linear increase (LI) when all the mean values for the four epididymal samples were significantly different from each other (*p*<0.05), expressed as increase (I) or decrease (D) when at least one of the mean values was not significantly different from the others, and intermediate (inter.) when at least one value was different. In the first column, *m*/*z* values in green correspond to *m*/*z* peaks observed only in IC analysis (a total of 135*m*/*z*). Rows with color in IC, SD and ID columns correspond to specific peaks for the sample preparation.

**A** list of endogenous biomolecules identified by top-down MS is shown in Supplementary Table 2. This table shows the raw file name associated to the NCBInr accession number, the gene name, the protein description, the location, the characterized post-translational modifications, the number of *b* ions, *y* ions and total ions, the delta mass (Da and ppm) between the ProSight theoretical mass *M* and the mass *M* observed by nano-ESI-FTMS, the mass *M* (Da) observed by nano-ESI-FTMS, the ProSight theoretical mass *M* (Da), the ProSight PDE score, the *E*-value identification probability, the *p* score, the precursor *m*/*z* and mass type with corresponding charge state (*z*), the fragmentation mode, the database used for identification, the signal/noise, the ProSight search type mode, the precursor mass type, the precursor mass tolerance (Da or ppm), the fragment mass type, the fragment mass tolerance (ppm), the delta *M* mode and disulfide activation/deactivation, the minimum of matching fragment between observed and theoretical fragmentation mass spectra, the include modified forms activation/deactivation, the sequence, the calculated theoretical average and monoisotopic mass [*M*+*H*]^+^ (Da) and the mass [*M*+*H*]^+^ (Da) observed previously by MALDI-MS.

## 2 Materials and methods: organ sampling and sperm preparation

**Fig. 1:**
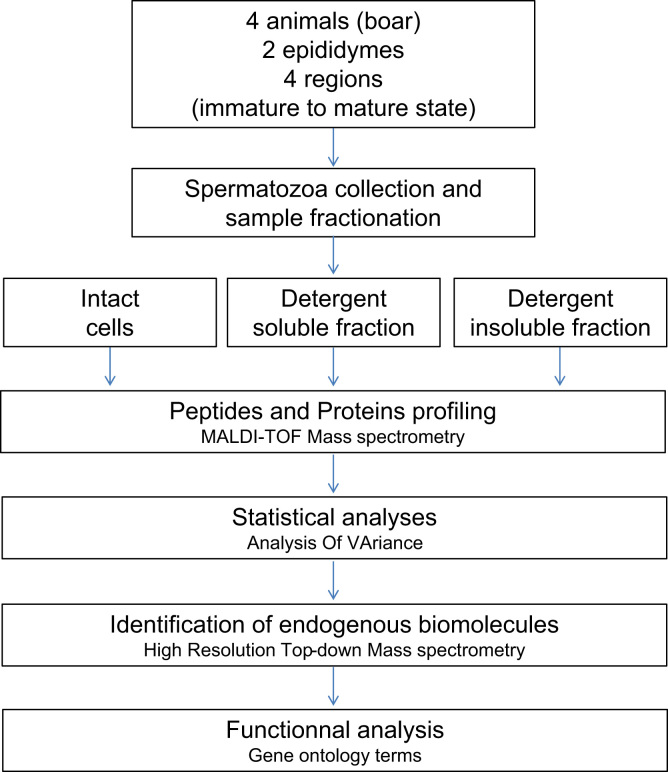
Experimental design.

The epididymides were collected from four one-year-old adult boars. The luminal contents of the tubules of four epididymal regions (E2, E4, E6 and E9) were collected by micro perfusion [2]. Spermatozoa were isolated by centrifugation. The pellets were washed once with PBS (140 mM NaCl, 15 mM KCl, 7 mM Na_2_HPO_4_, 1.5 mM KH_2_PO_4_, pH 7.4), centrifuged and resuspended in PBS. To remove protein contamination, the spermatozoa were centrifuged on 40% Percoll in PBS. The pellets were washed again with PBS and resuspended in 20 mM Tris–HCl, pH 6.8, and 260 mM sucrose (Tris–sucrose buffer (TS)) (Fig. 1).

## 3 Whole and fractionated cell preparations

For whole cell analysis, about 2×10^6^ spermatozoa were spotted for each epididymal region onto the MALDI sample probe and immediately mixed with a sinapinic acid matrix solution.

For fractionated cell preparations, sperm suspensions in TS buffer were centrifuged and about 1×10^7^ spermatozoa were resuspended in 1 volume of 2% octyl-β-D-1-thioglucopyrannoside in 20 mM Tris–HCl, pH 7, with 2 mM protease inhibitors (2 mM 4-(2-aminoethyl) benzenesulfonyl fluoride hydrochloride, 0.3 μM aprotinine, 130 μM bestatine hydrochloride, 14 μM E-64, 1 mM EDTA, 0.9 μM leupeptine hemisulfate and 1 mM phenylmethanesulfonyl fluoride). The mixture was agitated at 4 °C for 1 h and centrifuged. Supernatants corresponding to enriched membrane and cytosolic proteins (detergent-soluble extract (SD)) were dried and resuspended in TFA 0.1%. The pellet (detergent-insoluble extract (ID)), corresponding to nuclei, mitochondria and cytoskeleton, was washed with TS and centrifuged.

## 4 MALDI-TOF profiling of whole sperm cells and sub-cellular fractions

All samples were analyzed by a MALDI-TOF mass spectrometer (Waters Corporation, Micromass Ltd., Manchester, UK) operating in positive linear mode as previously described [1]. Spectral profiles were collected in the 2000–20,000*m*/*z* mass range. Data processing was performed using MassLynx^™^ 4.0 software. To increase mass accuracy, internal calibration was performed. Thus, the major unknown 6797*m*/*z* constant was used as “lock mass” for all spectra.

Intact spermatozoa and corresponding detergent-soluble and -insoluble extracts obtained from 4 epididymal regions (E2, E4, E6 and E9) of both epididymes from 4 animals were analyzed by MALDI-TOF MS, with 8 replicates for each of the three sample preparations. A total of 768 spectra were generated in this study. The spectra were analyzed by Progenesis MALDI^™^ 1.2 software (NonLinear Dynamics) as previously described [1]. After alignment of all spectra, a total of 253*m*/*z* peaks were detected (Supplementary Table 1). A total of 135*m*/*z* molecular species were characterized by Intact Cell MALDI-MS and 118*m*/*z* were newly observed by MALDI MS from SD and ID fractions.

## 5 Quantitative analysis linked to the sperm maturation process

In order to characterize peak differences between epididymal spermatozoa, the intensity of each normalized peak was subjected to one way analysis of variance with Progenesis (factor epididymal regions) and to three-way analysis of variance with R software (factors being animals, epididymis, epididymal regions, the replicate interactions being the residuals), as previously described [1]. All peak signals with a differential fold-change greater than two average values of normalized intensity and a *p* value<0.05 were selected (Supplementary Table 1). Thus 89*m*/*z* peaks were retained for intact cells (IC), 112*m*/*z* peaks for detergent-soluble extracts (SD) and 59*m*/*z* peaks for detergent-insoluble extracts (ID) for a total of 172 unique *m*/*z* peaks (Supplementary Table 1).

## 6 Top-down mass spectrometry

Identification of peptidoforms and proteoforms (endogenous species) was achieved by acquiring nano-ESI tandem high resolution mass spectrometry (MS and MS/MS). Molecular species were previously extracted with 1% and 5% formic acid from IC (region 2 or 9) or from ID fraction (region 9), desalted and concentrated using ZipTip C4 (Millipore Corporation, Billerica, MA). Eluted peptides and proteins were directly analyzed using a LTQ Orbitrap Velos mass spectrometer (Thermo Fisher Scientific, Germany) operating in positive mode, as previously described (1). Data were acquired using Xcalibur software v2.1 (Thermo Fisher Scientific, San Jose, CA). All analyses were performed manually using a high-high strategy, meaning that a MS spectrum in the 400–2000*m*/*z* mass range was followed by a MS/MS spectrum obtained by High energy Collisional Dissociation (HCD). Thus 412*m*/*z* corresponding to 217 non-redundant molecular ions were selected to induce HCD fragmentation.

Identification and structural characterization were performed using ProSight PC software 2.0 (Thermo Scientific, San Jose). Raw data files were processed by THRASH (signal/noise: 2–3), and data were compared to a simple annotated “*Sus scrofa*” house database generated from NCBInr using Proteome Discoverer (Thermo Fisher Scientific). Automated searches were performed using the “Absolute Mass and Biomarker” search options. The mass tolerances were set at 5 ppm for the monoisotopic precursors, 5 Da for the average precursor and 15 ppm for fragment ions mass tolerance. Disulfide modifications and N-terminal post-translation modifications (acetylation and initial methionine cleavage) were activated. Post-translational modifications such as phosphorylation and disulfide bridges were confirmed using the manual Single Protein mode. Proposed sequences with *E*-value<1×10^−2^ were considered positively identified with a minimum of 10 matching fragment ions. The data were deposited with the ProteomeXchange Consortium (http://proteomecentral.proteomexchange.org) via the PRIDE partner repository [3,4] with the dataset identifier PXD001303.

A total of 62*m*/*z* were identified and attributed to 32 different molecular species corresponding to three intact and whole proteins and 58 peptides from 29 proteins (Supplementary Table 2). Forty-five of these peptides presented tryptic or semi-tryptic cleavages, suggesting protease activities by trypsin-like or kallicrein enzymes (Supplementary Table 2).

**Fig. 2:**
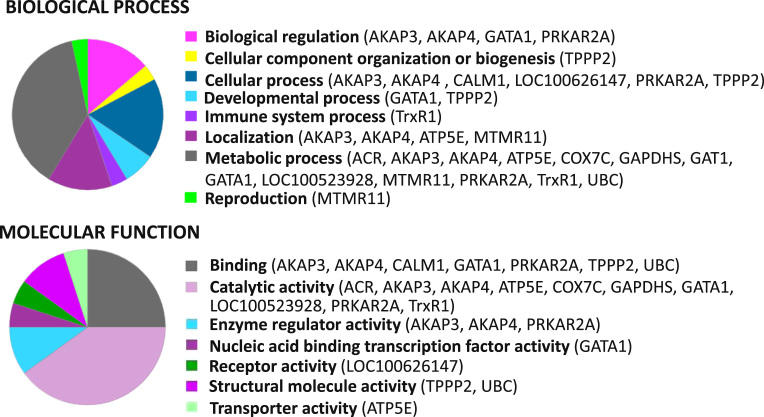
Classification of identified molecular species based on biological process and molecular function using PANTHER classification system.

Gene symbols were mapped for peptidoforms and proteoforms identified by top-down MS, and analyzed using the online PANTHER classification system (database version 9.0; http://www.pantherdb.org/) [5] (Fig. 2). Go terms from the Biological Process and the Molecular Function domains were considered. The background dataset for the analysis was the *Homo sapiens* and *Sus scrofa* genomes.

## Conflict of interest

The authors declare that they have no conflicts of interest.

## Footnotes

Appendix A Supplementary data associated with this article can be found in the online version at 10.1016/j.dib.2014.10.003.
