# Supplementary material for: Data in support of peptidomic analysis of spermatozoa during epididymal maturation
Source: Data Brief. 2014 Nov 6;1:79–84. doi: 10.1016/j.dib.2014.10.003 (PMC4459559; doi:10.1016/j.dib.2014.10.003)
Supplement: Supplementary file 1 — Supplementary data [file mmc1.zip › Table 1.docx]

Table 1

|  | | INTACT CELLS (IC) | | | | | | DETERGENT EXTRACTS (SD) | | | | | | INSOLUBLE DETERGENT EXTRACTS (ID) | | | | | |
| --- | --- | --- | --- | --- | --- | --- | --- | --- | --- | --- | --- | --- | --- | --- | --- | --- | --- | --- | --- |
| M/Z | Identification. | IC2-IC9 | Max | Min | Fold | Index variation | Variation | SD2-SD9 | Max | Min | Fold | Index variation | Variation | ID2-ID9 | Max | Min | Fold | Index variation | Variation |
| 2030.87 | **LOC100626147** | -917.3 | 1197.5 | 280.2 | 4.3 | 3919.7 | LI |  |  |  |  |  |  |  |  |  |  |  |  |
| 2086.08 |  |  |  |  |  |  |  |  |  |  |  |  |  |  |  |  |  |  |  |
| 2112.74 |  |  |  |  |  |  |  | -229.6 | 443.8 | 214.2 | 2.1 | 475.6 | I |  |  |  |  |  |  |
| 2140.48 |  |  |  |  |  |  |  | -445.2 | 765.7 | 320.5 | 2.4 | 1063.5 | I |  |  |  |  |  |  |
| 2157.48 | **ACRBP** | -12745.0 | 13437.2 | 692.2 | 19.4 | 247423.3 | LI | -26872.4 | 38023.1 | 11150.8 | 3.4 | 91632.4 | LI | -20432.2 | 15965.3 | 36397.4 | 2.3 | 46581.1 | I |
| 2172.98 | **ACRBP** | -532.8 | 705.6 | 172.8 | 4.1 | 2175.8 | I |  |  |  |  |  |  |  |  |  |  |  |  |
| 2188.48 | **LOC100626147** | -499.3 | 691.5 | 192.2 | 3.6 | 1796.1 | LI | -418.8 | 700.7 | 281.9 | 2.5 | 1041.0 | I |  |  |  |  |  |  |
| 2199.77 |  | -670.3 | 900.7 | 230.4 | 3.9 | 2620.6 | LI | -1141.2 | 1559.2 | 418.0 | 3.7 | 4256.7 | LI |  |  |  |  |  |  |
| 2220.03 |  |  |  |  |  |  |  |  |  |  |  |  |  | 180.9 | 166.3 | 347.3 | 2.1 | 377.8 | D |
| 2259.20 |  |  |  |  |  |  |  |  |  |  |  |  |  |  |  |  |  |  |  |
| 2271.44 |  |  |  |  |  |  |  |  |  |  |  |  |  |  |  |  |  |  |  |
| 2301.76 | **AKAP3** |  |  |  |  |  |  |  |  |  |  |  |  |  |  |  |  |  |  |
| 2336.17 |  | -372.6 | 628.2 | 255.6 | 2.5 | 915.6 | LI |  |  |  |  |  |  |  |  |  |  |  |  |
| 2363.37 |  | -460.1 | 688.6 | 228.5 | 3.0 | 1386.4 | I |  |  |  |  |  |  | -1000.8 | 987.3 | 2021.9 | 2.0 | 2049.3 | I |
| 2381.59 |  |  |  |  |  |  |  | -707.4 | 1275.8 | 568.4 | 2.2 | 1587.7 | I | -717.0 | 643.4 | 1360.4 | 2.1 | 1516.0 | I |
| 2402.67 |  |  |  |  |  |  |  |  |  |  |  |  |  | 407.0 | 504.1 | 1187.4 | 2.4 | 958.7 | D |
| 2417.90 |  |  |  |  |  |  |  |  |  |  |  |  |  |  |  |  |  |  |  |
| 2434.49 |  |  |  |  |  |  |  |  |  |  |  |  |  |  |  |  |  |  |  |
| 2453.39 | **AKAP4** | -455.7 | 712.6 | 256.9 | 2.8 | 1264.4 | LI |  |  |  |  |  |  |  |  |  |  |  |  |
| 2470.80 | **AKAP4** | -223.9 | 405.1 | 181.2 | 2.2 | 500.4 | I |  |  |  |  |  |  |  |  |  |  |  |  |
| 2492.15 |  |  |  |  |  |  |  | 346.9 | 496.6 | 149.7 | 3.3 | 1150.3 | D |  |  |  |  |  |  |
| 2530.77 |  |  |  |  |  |  |  | -396.5 | 724.4 | 327.9 | 2.2 | 875.8 | I |  |  |  |  |  |  |
| 2544.50 |  |  |  |  |  |  |  |  |  |  |  |  |  |  |  |  |  |  |  |
| 2579.73 |  |  |  |  |  |  |  |  |  |  |  |  |  |  |  |  |  |  |  |
| 2603.66 |  |  |  |  |  |  |  |  |  |  |  |  |  |  |  |  |  |  |  |
| 2630.72 |  |  |  |  |  |  |  |  |  |  |  |  |  |  |  |  |  |  |  |
| 2719.25 | **SPATA18** |  |  |  |  |  |  |  |  |  |  |  |  |  |  |  |  |  |  |
| 2735.43 |  |  |  |  |  |  |  | -92.6 | 469.9 | 218.8 | 2.1 | 198.8 | Inter. |  |  |  |  |  |  |
| 2773.24 |  | -270.6 | 464.8 | 190.4 | 2.4 | 660.7 | I |  |  |  |  |  |  |  |  |  |  |  |  |
| 2843.95 |  |  |  |  |  |  |  |  |  |  |  |  |  |  |  |  |  |  |  |
| 2892.64 | **C4H1orf56** | -294.4 | 508.7 | 214.3 | 2.4 | 698.8 | I |  |  |  |  |  |  | -515.0 | 887.6 | 1897.8 | 2.1 | 1101.2 | I |
| 2936.16 |  |  |  |  |  |  |  |  |  |  |  |  |  |  |  |  |  |  |  |
| 2948.94 |  |  |  |  |  |  |  | -168.4 | 335.9 | 167.5 | 2.0 | 337.6 | I |  |  |  |  |  |  |
| 2968.25 |  |  |  |  |  |  |  |  |  |  |  |  |  |  |  |  |  |  |  |
| 3002.93 |  | -596.7 | 791.6 | 194.9 | 4.1 | 2424.2 | LI | -1183.7 | 2240.8 | 1057.0 | 2.1 | 2509.4 | I |  |  |  |  |  |  |
| 3063.58 | **SPACA1** | -275.5 | 497.6 | 222.1 | 2.2 | 617.1 | I |  |  |  |  |  |  |  |  |  |  |  |  |
| 3090.91 |  |  |  |  |  |  |  | -171.3 | 669.1 | 294.8 | 2.3 | 388.7 | Inter. |  |  |  |  |  |  |
| 3117.97 |  |  |  |  |  |  |  |  |  |  |  |  |  |  |  |  |  |  |  |
| 3146.80 | **SPACA1** | -424.8 | 584.5 | 159.8 | 3.7 | 1553.9 | LI |  |  |  |  |  |  |  |  |  |  |  |  |
| 3159.32 |  |  |  |  |  |  |  |  |  |  |  |  |  | 385.7 | 350.3 | 736.1 | 2.1 | 810.5 | LD |
| 3202.97 |  |  |  |  |  |  |  | -301.3 | 530.8 | 229.4 | 2.3 | 697.1 | I |  |  |  |  |  |  |
| 3240.37 |  |  |  |  |  |  |  | -714.6 | 2651.7 | 807.3 | 3.3 | 2347.0 | Inter. |  |  |  |  |  |  |
| 3261.72 |  |  |  |  |  |  |  |  |  |  |  |  |  |  |  |  |  |  |  |
| 3314.21 |  |  |  |  |  |  |  |  |  |  |  |  |  |  |  |  |  |  |  |
| 3362.89 |  | -1033.0 | 1212.2 | 179.1 | 6.8 | 6991.4 | LI | -3252.7 | 3481.5 | 228.8 | 15.2 | 49489.0 | LI |  |  |  |  |  |  |
| 3390.77 | **ATP5E** |  |  |  |  |  |  | 3465.1 | 5203.6 | 1738.5 | 3.0 | 10371.2 | D | 719.2 | 693.0 | 1412.2 | 2.0 | 1465.5 | LD |
| 3443.13 |  |  |  |  |  |  |  | -324.3 | 1949.2 | 679.6 | 2.9 | 930.0 | Inter. |  |  |  |  |  |  |
| 3483.52 |  |  |  |  |  |  |  |  |  |  |  |  |  |  |  |  |  |  |  |
| 3501.47 |  |  |  |  |  |  |  |  |  |  |  |  |  |  |  |  |  |  |  |
| 3530.43 |  | -190.6 | 333.7 | 143.0 | 2.3 | 444.8 | I | -270.3 | 418.4 | 148.2 | 2.8 | 763.2 | I |  |  |  |  |  |  |
| 3603.73 |  |  |  |  |  |  |  |  |  |  |  |  |  |  |  |  |  |  |  |
| 3638.00 |  | -102.3 | 470.4 | 211.0 | 2.2 | 228.0 | Inter. | -135.4 | 964.9 | 226.4 | 4.3 | 576.9 | Inter. |  |  |  |  |  |  |
| 3654.45 |  |  |  |  |  |  |  |  |  |  |  |  |  | 140.4 | 141.4 | 301.7 | 2.1 | 299.6 | D |
| 3694.03 |  |  |  |  |  |  |  |  |  |  |  |  |  |  |  |  |  |  |  |
| 3760.12 |  | -201.3 | 319.4 | 118.0 | 2.7 | 544.8 | I |  |  |  |  |  |  |  |  |  |  |  |  |
| 3796.97 |  | -343.4 | 494.0 | 150.6 | 3.3 | 1126.4 | LI |  |  |  |  |  |  |  |  |  |  |  |  |
| 3818.19 |  |  |  |  |  |  |  |  |  |  |  |  |  | 231.6 | 178.2 | 409.8 | 2.3 | 532.7 | LD |
| 3869.59 |  | -210.5 | 367.0 | 156.5 | 2.3 | 493.8 | I |  |  |  |  |  |  |  |  |  |  |  |  |
| 3894.61 |  |  |  |  |  |  |  | -386.0 | 554.1 | 168.1 | 3.3 | 1272.5 | LI |  |  |  |  |  |  |
| 3912.97 |  |  |  |  |  |  |  | -125.5 | 655.7 | 221.4 | 3.0 | 371.8 | Inter. |  |  |  |  |  |  |
| 3956.49 |  |  |  |  |  |  |  | 185.6 | 389.6 | 183.9 | 2.1 | 393.3 | D |  |  |  |  |  |  |
| 3980.29 |  |  |  |  |  |  |  |  |  |  |  |  |  |  |  |  |  |  |  |
| 4017.68 |  | -169.3 | 328.6 | 159.3 | 2.1 | 349.3 | LI |  |  |  |  |  |  |  |  |  |  |  |  |
| 4039.31 |  |  |  |  |  |  |  |  |  |  |  |  |  |  |  |  |  |  |  |
| 4071.40 |  |  |  |  |  |  |  |  |  |  |  |  |  | 523.5 | 216.1 | 739.6 | 3.4 | 1791.8 | LD |
| 4125.12 |  |  |  |  |  |  |  |  |  |  |  |  |  | 440.8 | 274.9 | 809.2 | 2.9 | 1297.4 | D |
| 4136.40 |  |  |  |  |  |  |  |  |  |  |  |  |  | 314.5 | 231.2 | 599.1 | 2.6 | 814.8 | D |
| 4176.25 |  | -501.8 | 712.5 | 206.9 | 3.4 | 1728.0 | I |  |  |  |  |  |  |  |  |  |  |  |  |
| 4217.59 |  |  |  |  |  |  |  |  |  |  |  |  |  |  |  |  |  |  |  |
| 4287.08 |  |  |  |  |  |  |  |  |  |  |  |  |  | 172.1 | 159.1 | 331.2 | 2.1 | 358.1 | D |
| 4304.90 | **ACRV1** | 1151.5 | 1587.5 | 436.0 | 3.6 | 4193.0 | D | 302.4 | 739.8 | 278.9 | 2.7 | 802.0 | D |  |  |  |  |  |  |
| 4319.58 |  | 413.9 | 603.2 | 189.2 | 3.2 | 1319.5 | D | 623.5 | 915.4 | 282.9 | 3.2 | 2017.5 | D | -407.5 | 401.5 | 809.0 | 2.0 | 821.1 | I |
| 4388.53 |  |  |  |  |  |  |  |  |  |  |  |  |  |  |  |  |  |  |  |
| 4416.54 | **AKAP3** |  |  |  |  |  |  |  |  |  |  |  |  | 335.4 | 279.0 | 614.3 | 2.2 | 738.6 | LD |
| 4427.69 |  |  |  |  |  |  |  |  |  |  |  |  |  | 273.5 | 158.6 | 432.1 | 2.7 | 745.3 | LD |
| 4452.04 |  |  |  |  |  |  |  |  |  |  |  |  |  |  |  |  |  |  |  |
| 4491.61 |  | 895.2 | 1286.6 | 391.4 | 3.3 | 2942.2 | LD |  |  |  |  |  |  |  |  |  |  |  |  |
| 4513.10 | **C4H1orf56** |  |  |  |  |  |  |  |  |  |  |  |  |  |  |  |  |  |  |
| 4560.56 |  |  |  |  |  |  |  |  |  |  |  |  |  |  |  |  |  |  |  |
| 4604.62 |  | 618.6 | 779.7 | 161.2 | 4.8 | 2992.8 | LD | 413.7 | 558.2 | 144.4 | 3.9 | 1599.0 | LD | 483.9 | 122.3 | 606.2 | 5.0 | 2399.3 | LD |
| 4646.64 | **AKAP4** | 1454.1 | 2098.8 | 644.7 | 3.3 | 4733.5 | LD | 1273.6 | 2716.1 | 1157.7 | 2.3 | 2988.1 | LD | 2665.3 | 1135.2 | 3800.4 | 3.3 | 8923.1 | LD |
| 4665.27 |  |  |  |  |  |  |  | 63.9 | 105.7 | 41.8 | 2.5 | 161.8 | D | 527.1 | 124.1 | 651.2 | 5.2 | 2765.7 | LD |
| 4693.96 |  | 271.8 | 450.6 | 178.8 | 2.5 | 685.0 | LD |  |  |  |  |  |  | 126.7 | 125.3 | 252.0 | 2.0 | 254.9 | D |
| 4736.53 |  | -844.5 | 1901.4 | 459.1 | 4.1 | 3497.2 | I | 580.7 | 1463.3 | 508.1 | 2.9 | 1672.3 | LD | 1068.2 | 442.0 | 1510.2 | 3.4 | 3649.5 | LD |
| 4762.91 |  | 488.5 | 1108.6 | 189.3 | 5.9 | 2860.4 | LD | 598.1 | 905.3 | 142.3 | 6.4 | 3805.3 | D | 613.2 | 207.8 | 820.9 | 4.0 | 2422.4 | LD |
| 4817.85 |  |  |  |  |  |  |  |  |  |  |  |  |  | 359.9 | 227.2 | 606.0 | 2.7 | 959.6 | D |
| 4855.93 | **PRKAR2A** | 795.5 | 1016.3 | 220.9 | 4.6 | 3660.5 | LD | 411.3 | 842.1 | 310.8 | 2.7 | 1114.5 | LD |  |  |  |  |  |  |
| 4867.35 |  | 476.2 | 634.3 | 158.2 | 4.0 | 1909.8 | D |  |  |  |  |  |  |  |  |  |  |  |  |
| 4910.87 |  |  |  |  |  |  |  | -164.1 | 294.8 | 130.7 | 2.3 | 370.2 | I |  |  |  |  |  |  |
| 4931.27 |  |  |  |  |  |  |  |  |  |  |  |  |  |  |  |  |  |  |  |
| 4967.44 |  |  |  |  |  |  |  |  |  |  |  |  |  | 184.3 | 131.6 | 316.0 | 2.4 | 442.5 | D |
| 4993.96 | **AKAP4** | 34.9 | 659.1 | 312.7 | 2.1 | 73.5 | Inter. | 854.4 | 2745.4 | 1038.2 | 2.6 | 2259.3 | D | 2444.9 | 1030.5 | 3674.5 | 3.6 | 8718.0 | D |
| 5008.64 | **AKAP4** | 76.6 | 647.6 | 202.4 | 3.2 | 245.2 | LD | -661.6 | 1422.2 | 363.6 | 3.9 | 2587.4 | I | 428.4 | 207.1 | 710.0 | 3.4 | 1468.6 | LD |
| 5053.79 | **PRKAR2A** |  |  |  |  |  |  | 207.4 | 417.8 | 159.4 | 2.6 | 543.4 | D |  |  |  |  |  |  |
| 5072.69 | **AKAP4** |  |  |  |  |  |  |  |  |  |  |  |  |  |  |  |  |  |  |
| 5107.24 |  | -3869.1 | 4203.1 | 334.0 | 12.6 | 48682.1 | LI | -1685.6 | 2371.6 | 686.0 | 3.5 | 5827.4 | LI |  |  |  |  |  |  |
| 5159.59 |  | -165.5 | 320.6 | 155.1 | 2.1 | 342.1 | I | -447.6 | 624.9 | 158.8 | 3.9 | 1761.6 | I |  |  |  |  |  |  |
| 5202.29 |  |  |  |  |  |  |  | 902.0 | 1493.8 | 591.8 | 2.5 | 2277.0 | LD | 270.6 | 176.1 | 446.7 | 2.5 | 686.6 | LD |
| 5229.63 |  | 356.3 | 462.6 | 106.3 | 4.4 | 1550.5 | LD |  |  |  |  |  |  |  |  |  |  |  |  |
| 5267.57 |  |  |  |  |  |  |  |  |  |  |  |  |  |  |  |  |  |  |  |
| 5295.85 |  |  |  |  |  |  |  |  |  |  |  |  |  |  |  |  |  |  |  |
| 5313.81 |  | -467.8 | 837.1 | 109.9 | 7.6 | 3562.1 | Inter. |  |  |  |  |  |  | -19.2 | 284.7 | 709.6 | 2.5 | 47.8 | Inter. |
| 5381.80 |  | -139.9 | 1642.8 | 321.0 | 5.1 | 716.0 | Inter. | -256.9 | 1571.1 | 342.3 | 4.6 | 1178.9 | Inter. |  |  |  |  |  |  |
| 5409.41 |  |  |  |  |  |  |  | 156.0 | 770.3 | 228.4 | 3.4 | 526.1 | D | 411.8 | 183.5 | 595.3 | 3.2 | 1336.0 | D |
| 5457.82 | **COX7C** |  |  |  |  |  |  |  |  |  |  |  |  |  |  |  |  |  |  |
| 5520.10 |  | 1018.8 | 1185.8 | 167.0 | 7.1 | 7232.7 | LD | 442.9 | 591.3 | 148.4 | 4.0 | 1765.4 | LD | 301.7 | 108.7 | 410.4 | 3.8 | 1139.3 | LD |
| 5565.93 |  | 3172.0 | 3452.2 | 280.2 | 12.3 | 39076.5 | LD | 702.5 | 1139.7 | 361.7 | 3.2 | 2213.6 | D | 613.2 | 424.0 | 1058.4 | 2.5 | 1530.5 | D |
| 5583.61 |  | 336.6 | 406.5 | 69.9 | 5.8 | 1957.3 | LD | 223.6 | 313.6 | 90.0 | 3.5 | 778.9 | D | 417.9 | 80.9 | 502.1 | 6.2 | 2593.4 | LD |
| 5618.83 | **ATP5E** |  |  |  |  |  |  | 794.8 | 1149.5 | 354.7 | 3.2 | 2575.7 | LD |  |  |  |  |  |  |
| 5663.57 |  | 174.7 | 306.2 | 131.5 | 2.3 | 406.9 | D |  |  |  |  |  |  |  |  |  |  |  |  |
| 5703.69 |  | -64.7 | 272.8 | 129.0 | 2.1 | 136.8 | Inter. | 668.2 | 1295.0 | 581.3 | 2.2 | 1488.7 | D |  |  |  |  |  |  |
| 5748.02 |  |  |  |  |  |  |  | 64.4 | 305.1 | 142.5 | 2.1 | 137.9 | D |  |  |  |  |  |  |
| 5782.29 |  | 1779.5 | 2550.1 | 770.6 | 3.3 | 5888.7 | D |  |  |  |  |  |  | 948.1 | 655.2 | 1603.3 | 2.4 | 2319.9 | LD |
| 5816.83 |  | 546.3 | 647.6 | 101.3 | 6.4 | 3491.1 | LD | 113.8 | 206.3 | 92.5 | 2.2 | 254.0 | D | 199.3 | 162.3 | 370.0 | 2.3 | 454.4 | D |
| 5861.03 |  | 586.6 | 1219.7 | 341.8 | 3.6 | 2092.8 | Inter. | 487.8 | 756.0 | 210.9 | 3.6 | 1749.0 | D |  |  |  |  |  |  |
| 6008.17 |  | 173.9 | 270.9 | 97.0 | 2.8 | 485.4 | LD | 128.7 | 234.3 | 105.5 | 2.2 | 285.7 | D |  |  |  |  |  |  |
| 6055.22 |  | -110.6 | 239.7 | 98.6 | 2.4 | 269.0 | I | 277.9 | 369.4 | 91.5 | 4.0 | 1122.0 | D | 242.7 | 82.2 | 325.0 | 4.0 | 959.6 | D |
| 6074.53 |  |  |  |  |  |  |  | 121.9 | 221.6 | 99.6 | 2.2 | 271.2 | D |  |  |  |  |  |  |
| 6096.70 |  | -1291.6 | 2172.6 | 417.4 | 5.2 | 6722.3 | Inter. | -1697.4 | 2197.9 | 500.5 | 4.4 | 7454.0 | I | -749.7 | 124.1 | 886.8 | 7.1 | 5358.7 | I |
| 6111.93 |  |  |  |  |  |  |  |  |  |  |  |  |  |  |  |  |  |  |  |
| 6145.25 | **LOC100626147** | -739.1 | 1138.0 | 139.0 | 8.2 | 6053.4 | I |  |  |  |  |  |  | -260.5 | 519.8 | 1072.7 | 2.1 | 537.4 | D |
| 6228.20 | **ZPBP1** |  |  |  |  |  |  | 1426.5 | 2306.0 | 879.5 | 2.6 | 3740.4 | LD |  |  |  |  |  |  |
| 6252.54 |  | -191.7 | 617.3 | 123.6 | 5.0 | 957.2 | Inter. |  |  |  |  |  |  |  |  |  |  |  |  |
| 6295.38 |  |  |  |  |  |  |  |  |  |  |  |  |  |  |  |  |  |  |  |
| 6309.93 |  | -314.0 | 546.8 | 232.8 | 2.3 | 737.3 | I | -182.1 | 440.8 | 210.7 | 2.1 | 381.0 | I |  |  |  |  |  |  |
| 6325.30 |  | -518.0 | 772.5 | 254.5 | 3.0 | 1572.6 | I |  |  |  |  |  |  |  |  |  |  |  |  |
| 6384.45 |  |  |  |  |  |  |  |  |  |  |  |  |  |  |  |  |  |  |  |
| 6409.07 |  | -132.1 | 524.1 | 192.8 | 2.7 | 359.0 | Inter. |  |  |  |  |  |  |  |  |  |  |  |  |
| 6427.29 |  | 222.3 | 334.5 | 112.2 | 3.0 | 663.0 | D |  |  |  |  |  |  |  |  |  |  |  |  |
| 6467.82 |  | -308.3 | 505.2 | 196.9 | 2.6 | 791.2 | LI | 218.8 | 451.6 | 217.5 | 2.1 | 454.4 | D | -118.0 | 131.1 | 264.2 | 2.0 | 237.9 | Inter. |
| 6495.83 |  |  |  |  |  |  |  |  |  |  |  |  |  | 91.1 | 110.1 | 223.1 | 2.0 | 184.6 | Inter. |
| 6532.00 |  |  |  |  |  |  |  |  |  |  |  |  |  |  |  |  |  |  |  |
| 6563.28 |  |  |  |  |  |  |  |  |  |  |  |  |  |  |  |  |  |  |  |
| 6591.57 |  |  |  |  |  |  |  |  |  |  |  |  |  |  |  |  |  |  |  |
| 6656.84 |  |  |  |  |  |  |  |  |  |  |  |  |  |  |  |  |  |  |  |
| 6685.81 |  |  |  |  |  |  |  | 330.8 | 428.1 | 97.3 | 4.4 | 1455.3 | D |  |  |  |  |  |  |
| 6713.01 |  | -899.7 | 1480.7 | 109.0 | 13.6 | 12225.4 | Inter. | -252.4 | 868.5 | 255.9 | 3.4 | 856.4 | Inter. | -994.0 | 167.5 | 1161.5 | 6.9 | 6891.8 | I |
| 6797.18 |  |  |  |  |  |  |  | 8097.9 | 11145.9 | 3048.0 | 3.7 | 29612.2 | D |  |  |  |  |  |  |
| 6833.90 |  | 177.0 | 237.4 | 60.4 | 3.9 | 695.3 | D | 78.3 | 141.1 | 62.8 | 2.2 | 175.9 | D |  |  |  |  |  |  |
| 6854.98 |  |  |  |  |  |  |  |  |  |  |  |  |  |  |  |  |  |  |  |
| 6897.82 |  |  |  |  |  |  |  |  |  |  |  |  |  |  |  |  |  |  |  |
| 6977.37 |  |  |  |  |  |  |  | 128.0 | 211.5 | 83.5 | 2.5 | 324.3 | D |  |  |  |  |  |  |
| 7004.16 |  |  |  |  |  |  |  | 290.2 | 412.7 | 122.5 | 3.4 | 977.9 | D |  |  |  |  |  |  |
| 7021.16 |  |  |  |  |  |  |  | 302.4 | 473.1 | 170.7 | 2.8 | 837.8 | D |  |  |  |  |  |  |
| 7067.26 |  |  |  |  |  |  |  | -38.0 | 89.3 | 43.8 | 2.0 | 77.4 | I | -151.8 | 91.3 | 247.3 | 2.7 | 411.1 | I |
| 7108.60 |  |  |  |  |  |  |  |  |  |  |  |  |  |  |  |  |  |  |  |
| 7158.37 |  |  |  |  |  |  |  | -234.7 | 622.3 | 287.6 | 2.2 | 507.8 | I |  |  |  |  |  |  |
| 7193.46 |  |  |  |  |  |  |  |  |  |  |  |  |  |  |  |  |  |  |  |
| 7232.76 |  | 151.2 | 258.1 | 106.8 | 2.4 | 365.4 | LD | 487.6 | 778.3 | 140.1 | 5.6 | 2708.1 | D |  |  |  |  |  |  |
| 7269.61 |  |  |  |  |  |  |  |  |  |  |  |  |  |  |  |  |  |  |  |
| 7310.27 |  |  |  |  |  |  |  | 113.1 | 219.7 | 106.5 | 2.1 | 233.2 | D |  |  |  |  |  |  |
| 7368.21 |  |  |  |  |  |  |  |  |  |  |  |  |  |  |  |  |  |  |  |
| 7403.29 |  | 275.7 | 431.7 | 156.0 | 2.8 | 762.8 | LD | 185.7 | 323.3 | 137.6 | 2.3 | 436.3 | LD |  |  |  |  |  |  |
| 7431.03 |  |  |  |  |  |  |  |  |  |  |  |  |  | -141.2 | 109.7 | 251.0 | 2.3 | 323.0 | I |
| 7457.01 |  |  |  |  |  |  |  | -192.5 | 289.9 | 97.4 | 3.0 | 573.0 | I |  |  |  |  |  |  |
| 7501.61 |  |  |  |  |  |  |  | -51.1 | 109.2 | 42.3 | 2.6 | 132.2 | I |  |  |  |  |  |  |
| 7521.74 |  |  |  |  |  |  |  | -744.6 | 971.9 | 197.6 | 4.9 | 3662.2 | LI |  |  |  |  |  |  |
| 7621.69 |  | -396.4 | 650.3 | 253.9 | 2.6 | 1015.3 | LI |  |  |  |  |  |  | -377.9 | 196.8 | 574.7 | 2.9 | 1103.5 | LI |
| 7661.40 |  | -253.1 | 360.0 | 106.9 | 3.4 | 852.4 | LI | -622.3 | 870.7 | 248.4 | 3.5 | 2181.0 | I | -257.5 | 131.6 | 389.1 | 3.0 | 761.0 | Inter. |
| 7679.62 |  |  |  |  |  |  |  |  |  |  |  |  |  |  |  |  |  |  |  |
| 7705.46 |  | 174.9 | 246.4 | 71.5 | 3.4 | 602.7 | D |  |  |  |  |  |  |  |  |  |  |  |  |
| 7736.19 |  |  |  |  |  |  |  |  |  |  |  |  |  |  |  |  |  |  |  |
| 7784.34 |  |  |  |  |  |  |  |  |  |  |  |  |  |  |  |  |  |  |  |
| 7865.25 |  |  |  |  |  |  |  | -265.6 | 451.3 | 180.4 | 2.5 | 664.2 | I |  |  |  |  |  |  |
| 7898.29 |  |  |  |  |  |  |  | 221.5 | 339.1 | 117.6 | 2.9 | 638.5 | LD |  |  |  |  |  |  |
| 7945.48 |  |  |  |  |  |  |  |  |  |  |  |  |  |  |  |  |  |  |  |
| 8015.38 |  | 152.5 | 234.8 | 82.3 | 2.9 | 435.2 | LD |  |  |  |  |  |  |  |  |  |  |  |  |
| 8045.30 |  |  |  |  |  |  |  |  |  |  |  |  |  |  |  |  |  |  |  |
| 8068.42 |  |  |  |  |  |  |  |  |  |  |  |  |  |  |  |  |  |  |  |
| 8099.15 |  |  |  |  |  |  |  |  |  |  |  |  |  |  |  |  |  |  |  |
| 8153.47 |  |  |  |  |  |  |  |  |  |  |  |  |  |  |  |  |  |  |  |
| 8190.95 |  |  |  |  |  |  |  | 161.9 | 247.4 | 85.4 | 2.9 | 468.8 | D |  |  |  |  |  |  |
| 8242.08 |  |  |  |  |  |  |  | 298.2 | 504.3 | 206.2 | 2.4 | 729.5 | LD |  |  |  |  |  |  |
| 8300.01 |  |  |  |  |  |  |  |  |  |  |  |  |  |  |  |  |  |  |  |
| 8371.68 |  |  |  |  |  |  |  | -179.7 | 262.0 | 82.3 | 3.2 | 572.4 | I |  |  |  |  |  |  |
| 8392.89 |  |  |  |  |  |  |  | 88.7 | 165.7 | 77.1 | 2.2 | 190.7 | D |  |  |  |  |  |  |
| 8443.07 |  |  |  |  |  |  |  |  |  |  |  |  |  |  |  |  |  |  |  |
| 8468.91 |  |  |  |  |  |  |  |  |  |  |  |  |  |  |  |  |  |  |  |
| 8511.75 |  |  |  |  |  |  |  |  |  |  |  |  |  |  |  |  |  |  |  |
| 8562.47 | **UBC** |  |  |  |  |  |  | 765.6 | 1324.7 | 500.8 | 2.6 | 2025.4 | D |  |  |  |  |  |  |
| 8641.89 |  |  |  |  |  |  |  |  |  |  |  |  |  |  |  |  |  |  |  |
| 8670.04 |  |  |  |  |  |  |  |  |  |  |  |  |  |  |  |  |  |  |  |
| 8761.42 |  |  |  |  |  |  |  |  |  |  |  |  |  |  |  |  |  |  |  |
| 8899.05 |  |  |  |  |  |  |  | -232.1 | 257.5 | 25.4 | 10.1 | 2351.9 | I |  |  |  |  |  |  |
| 8928.15 |  |  |  |  |  |  |  | 290.5 | 509.8 | 197.4 | 2.6 | 750.3 | D |  |  |  |  |  |  |
| 8992.47 |  |  |  |  |  |  |  |  |  |  |  |  |  |  |  |  |  |  |  |
| 9083.99 |  |  |  |  |  |  |  |  |  |  |  |  |  |  |  |  |  |  |  |
| 9148.45 |  | 530.8 | 614.3 | 83.5 | 7.4 | 3905.1 | LD |  |  |  |  |  |  | 159.3 | 132.5 | 292.7 | 2.2 | 352.0 | D |
| 9215.63 |  |  |  |  |  |  |  | 180.4 | 348.5 | 168.1 | 2.1 | 374.1 | D |  |  |  |  |  |  |
| 9264.99 |  |  |  |  |  |  |  | -491.5 | 530.5 | 38.9 | 13.6 | 6701.1 | LI |  |  |  |  |  |  |
| 9368.07 |  |  |  |  |  |  |  | 263.1 | 441.5 | 170.5 | 2.6 | 681.2 | D |  |  |  |  |  |  |
| 9387.39 |  |  |  |  |  |  |  | 146.9 | 261.3 | 114.4 | 2.3 | 335.4 | LD |  |  |  |  |  |  |
| 9413.36 |  |  |  |  |  |  |  | -128.8 | 360.9 | 167.8 | 2.2 | 277.0 | Inter. |  |  |  |  |  |  |
| 9492.78 |  |  |  |  |  |  |  | 93.2 | 182.0 | 88.8 | 2.0 | 190.9 | D |  |  |  |  |  |  |
| 9538.20 |  |  |  |  |  |  |  | 104.4 | 181.8 | 77.4 | 2.3 | 245.4 | D |  |  |  |  |  |  |
| 9577.50 |  | 1286.8 | 1451.3 | 164.5 | 8.8 | 11356.1 | LD | 1410.6 | 1616.7 | 206.1 | 7.8 | 11062.8 | LD |  |  |  |  |  |  |
| 9635.98 |  |  |  |  |  |  |  |  |  |  |  |  |  |  |  |  |  |  |  |
| 9692.55 |  |  |  |  |  |  |  | 963.3 | 1145.6 | 182.3 | 6.3 | 6053.5 | LD |  |  |  |  |  |  |
| 9731.98 |  |  |  |  |  |  |  | 240.8 | 367.0 | 104.6 | 3.5 | 845.2 | D |  |  |  |  |  |  |
| 9755.10 |  |  |  |  |  |  |  | 299.7 | 341.1 | 41.4 | 8.2 | 2468.8 | LD |  |  |  |  |  |  |
| 9803.92 |  | 316.7 | 462.4 | 145.7 | 3.2 | 1005.4 | LD | 565.6 | 1056.4 | 490.8 | 2.2 | 1217.5 | D |  |  |  |  |  |  |
| 9885.38 |  | -548.0 | 664.8 | 103.3 | 6.4 | 3527.5 | I | 1251.7 | 1412.8 | 161.1 | 8.8 | 10979.8 | LD | -420.2 | 386.6 | 806.8 | 2.1 | 876.8 | I |
| 10259.49 |  |  |  |  |  |  |  |  |  |  |  |  |  |  |  |  |  |  |  |
| 10269.69 |  |  |  |  |  |  |  |  |  |  |  |  |  |  |  |  |  |  |  |
| 10504.27 |  |  |  |  |  |  |  | 167.1 | 243.0 | 75.9 | 3.2 | 534.9 | D |  |  |  |  |  |  |
| 10546.56 |  |  |  |  |  |  |  |  |  |  |  |  |  |  |  |  |  |  |  |
| 10587.77 |  | 334.4 | 402.1 | 67.8 | 5.9 | 1984.9 | D | 302.1 | 365.8 | 63.7 | 5.7 | 1734.2 | D | 192.6 | 76.8 | 269.4 | 3.5 | 675.3 | D |
| 10646.38 |  |  |  |  |  |  |  |  |  |  |  |  |  |  |  |  |  |  |  |
| 10866.69 |  |  |  |  |  |  |  |  |  |  |  |  |  |  |  |  |  |  |  |
| 10904.49 |  | -105.0 | 213.1 | 95.7 | 2.2 | 233.9 | I |  |  |  |  |  |  | -84.0 | 101.3 | 203.9 | 2.0 | 169.2 | I |
| 10983.23 |  |  |  |  |  |  |  | 152.1 | 282.9 | 83.6 | 3.4 | 514.8 | D |  |  |  |  |  |  |
| 11181.50 |  | 305.5 | 389.3 | 83.7 | 4.6 | 1420.1 | LD |  |  |  |  |  |  |  |  |  |  |  |  |
| 11236.71 |  |  |  |  |  |  |  |  |  |  |  |  |  |  |  |  |  |  |  |
| 11260.51 |  | 182.1 | 216.1 | 34.0 | 6.4 | 1157.9 | D |  |  |  |  |  |  |  |  |  |  |  |  |
| 11331.64 |  | 135.4 | 195.2 | 59.8 | 3.3 | 441.6 | D |  |  |  |  |  |  |  |  |  |  |  |  |
| 11459.60 |  | -2724.7 | 3300.5 | 575.8 | 5.7 | 15616.9 | LI | -1805.4 | 2177.6 | 372.2 | 5.9 | 10563.3 | I | -2040.5 | 340.0 | 2380.5 | 7.0 | 14287.8 | LI |
| 11559.01 | **NCAPH2** |  |  |  |  |  |  |  |  |  |  |  |  |  |  |  |  |  |  |
| 11749.12 |  |  |  |  |  |  |  | -172.4 | 233.4 | 59.0 | 4.0 | 682.2 | I |  |  |  |  |  |  |
| 11782.03 |  |  |  |  |  |  |  | 83.0 | 181.2 | 82.3 | 2.2 | 182.8 | D |  |  |  |  |  |  |
| 11949.98 | **ACRV1** | -2341.5 | 2544.7 | 155.3 | 16.4 | 38355.2 | LI | -147.2 | 390.9 | 108.8 | 3.6 | 528.6 | I | -1436.5 | 104.8 | 1552.6 | 14.8 | 21290.5 | I |
| 11976.50 |  |  |  |  |  |  |  | 597.7 | 924.3 | 326.6 | 2.8 | 1691.5 | D |  |  |  |  |  |  |
| 12114.26 |  |  |  |  |  |  |  |  |  |  |  |  |  | 103.4 | 74.9 | 178.3 | 2.4 | 246.3 | D |
| 12149.21 |  |  |  |  |  |  |  | -115.7 | 338.7 | 159.9 | 2.1 | 245.1 | I |  |  |  |  |  |  |
| 12227.81 |  |  |  |  |  |  |  |  |  |  |  |  |  |  |  |  |  |  |  |
| 12302.19 |  |  |  |  |  |  |  |  |  |  |  |  |  | -146.7 | 85.8 | 232.6 | 2.7 | 397.6 | LI |
| 12433.97 |  |  |  |  |  |  |  |  |  |  |  |  |  |  |  |  |  |  |  |
| 12491.49 |  | -502.3 | 677.6 | 165.6 | 4.1 | 2055.5 | I | -80.0 | 156.3 | 62.8 | 2.5 | 199.0 | I | -284.2 | 118.8 | 423.9 | 3.6 | 1014.3 | I |
| 12542.63 |  |  |  |  |  |  |  |  |  |  |  |  |  |  |  |  |  |  |  |
| 12636.73 |  |  |  |  |  |  |  |  |  |  |  |  |  |  |  |  |  |  |  |
| 12724.17 |  | -118.4 | 230.0 | 97.8 | 2.4 | 278.2 | LI |  |  |  |  |  |  | -80.8 | 78.6 | 159.6 | 2.0 | 164.1 | I |
| 12786.59 |  |  |  |  |  |  |  |  |  |  |  |  |  |  |  |  |  |  |  |
| 12887.50 |  |  |  |  |  |  |  | -125.5 | 174.5 | 33.4 | 5.2 | 656.3 | I |  |  |  |  |  |  |
| 12942.98 |  |  |  |  |  |  |  | 912.7 | 1807.8 | 895.1 | 2.0 | 1843.4 | D | -26.4 | 107.6 | 220.9 | 2.1 | 54.3 | Inter. |
| 13005.94 |  | -196.2 | 294.2 | 97.9 | 3.0 | 589.3 | I |  |  |  |  |  |  |  |  |  |  |  |  |
| 13150.77 |  | -428.5 | 544.0 | 110.8 | 4.9 | 2103.7 | I |  |  |  |  |  |  | -253.2 | 77.8 | 336.9 | 4.3 | 1097.0 | I |
| 13634.08 |  | -437.4 | 622.3 | 184.9 | 3.4 | 1472.2 | LI |  |  |  |  |  |  | -131.9 | 94.6 | 234.1 | 2.5 | 326.5 | I |
| 13780.14 |  |  |  |  |  |  |  | -74.7 | 195.4 | 69.5 | 2.8 | 210.0 | I |  |  |  |  |  |  |
| 14113.99 |  | -240.3 | 342.6 | 102.2 | 3.4 | 805.3 | D | -147.8 | 207.9 | 55.2 | 3.8 | 556.8 | I | -143.2 | 91.7 | 237.7 | 2.6 | 371.4 | I |
| 14220.88 |  |  |  |  |  |  |  |  |  |  |  |  |  |  |  |  |  |  |  |
| 14325.32 |  |  |  |  |  |  |  | 538.9 | 1197.4 | 581.8 | 2.1 | 1109.1 | D |  |  |  |  |  |  |
| 14597.98 |  | -181.5 | 366.0 | 114.0 | 3.2 | 582.8 | D |  |  |  |  |  |  |  |  |  |  |  |  |
| 15036.27 |  | 103.5 | 180.6 | 77.1 | 2.3 | 242.4 | D | 214.9 | 333.8 | 118.9 | 2.8 | 603.1 | D |  |  |  |  |  |  |
| 15080.47 |  | -47.6 | 256.2 | 111.9 | 2.3 | 108.9 | Inter. |  |  |  |  |  |  |  |  |  |  |  |  |
| 15186.27 | **ACRBP** |  |  |  |  |  |  | 117.8 | 229.1 | 111.3 | 2.1 | 242.3 | D |  |  |  |  |  |  |
| 15579.83 |  |  |  |  |  |  |  |  |  |  |  |  |  |  |  |  |  |  |  |
| 15736.62 |  |  |  |  |  |  |  |  |  |  |  |  |  |  |  |  |  |  |  |
| 15799.72 |  |  |  |  |  |  |  |  |  |  |  |  |  |  |  |  |  |  |  |
| 15893.01 |  | -214.5 | 299.5 | 85.0 | 3.5 | 755.9 | I | -29.8 | 383.2 | 148.9 | 2.6 | 76.6 | I |  |  |  |  |  |  |
| 16505.92 |  |  |  |  |  |  |  |  |  |  |  |  |  |  |  |  |  |  |  |
| 16791.77 |  |  |  |  |  |  |  | 160.1 | 238.6 | 75.3 | 3.2 | 507.6 | D |  |  |  |  |  |  |
| 17147.93 |  | 41.5 | 296.3 | 127.4 | 2.3 | 96.5 | Inter. |  |  |  |  |  |  |  |  |  |  |  |  |
| 17520.54 |  | -177.7 | 324.5 | 122.2 | 2.7 | 471.8 | D |  |  |  |  |  |  |  |  |  |  |  |  |
| 18451.26 |  | 258.4 | 421.8 | 163.4 | 2.6 | 667.1 | LD | 132.8 | 257.8 | 125.0 | 2.1 | 273.8 | D |  |  |  |  |  |  |
